# Association between total available nutritional quality and food expenditure in Peruvian households, 2019-2020

**DOI:** 10.1590/0102-311XEN021923

**Published:** 2023-09-18

**Authors:** Karen Bonilla-Aguilar, Antonio Bernabe-Ortiz

**Affiliations:** 1 Universidad Cientifica del Sur, Lima, Perú.

**Keywords:** Nutritional Quality, Expenditure, Family Characteristics, Diet Surveys, Valor Nutritivo, Gasto, Composición Familiar, Encuestas sobre Dietas, Valor Nutritivo, Gasto, Características da Família, Inquéritos sobre Dietas

## Abstract

Evidence points to a direct relationship between nutritional quality and food expenditure. However, food expenditure is highly susceptible to changes, and nutritional quality of household food presents limited evidence. The aim of this study was to assess the relationship between nutritional quality available and total food expenditure in Peruvian households, and whether there were differences by area (urban and rural) and between years of the COVID-19 pandemic. For this, we used *Peru’s National Household Survey* (ENAHO) from 2019 and 2020. We assessed total food expenditure in US dollars per day, whereas household nutritional quality available was assessed based on dietary diversity and compliance with the household calorie requirements, percentage of food expenditure, and potential confounders. We used the Student’s t-test, analysis of variance (ANOVA), linear regression, and the Wald test to assess the interaction effect. Households with adequate total/partial nutritional quality available by area were found to spend, on average, USD 2.00 more in urban than in rural areas and, by year, they presented 7.1% more percentage of food expenditure in 2020 than in 2019. Despite associations existing between nutritional quality available and total food expenditure by year and study area, the effect modification was only present by study area. In multivariable model, households with adequate total/partial nutritional quality available consistently presented a lower total food expenditure by year, with a lower total food expenditure in urban areas. An inverse relationship was found between nutritional quality available and total food expenditure, in contrast to the direct relationship of studies assessing dietary cost and nutritional quality. Our results reflect the nutritional deficit in the food purchases of Peruvian households.

## Resumen

Las evidencian existentes indican una relación directa entre la calidad nutricional y el gasto con alimentos. Sin embargo, los gastos son muy susceptibles a los cambios. Por otro lado, hay pruebas limitadas sobre la calidad nutricional de los alimentos domésticos disponibles. El objetivo de este estudio fue evaluar la relación entre la calidad nutricional disponible y el gasto total en alimentos en los hogares peruanos, y si hubo diferencias por área (urbana y rural) y entre los años de la pandemia del COVID-19. Para ello, se utilizó la *Encuesta Nacional de Hogares del Perú* (ENAHO) de 2019 y 2020. Se evalúo el gasto total en alimentos en dólares estadounidenses por día, la calidad nutricional disponible por hogar basado en la diversidad dietética y el cumplimiento de los requisitos calóricos por hogar, teniendo en cuenta el porcentaje de gasto alimentario y los posibles factores de confusión. La prueba t de Student, el análisis de varianza (ANOVA), la regresión lineal y la prueba de Wald se utilizaron para evaluar el efecto de interacción. Se encontró que los hogares con la calidad nutricional disponible total/parcial adecuada por área gastan en promedio USD 2,00 más en áreas urbanas que en áreas rurales, y que por año tuvieron el porcentaje de gasto alimentario 7,1% más grande en 2020 que en 2019. Si bien hubo una asociación entre calidad nutricional disponible y gasto total en alimentos por año y área de estudio, la modificación del efecto solo estuvo presente por área de estudio. En el modelo multivariable, los hogares con la calidad nutricional disponible total/parcial adecuada tuvieron consistentemente un gasto total en alimentos más bajo por año, de la misma manera que en áreas urbanas. Se encontró una relación inversa entre calidad nutricional disponible y gasto total en alimentos en contraste con la relación directa de los estudios que evaluaron el costo de la dieta y la calidad nutricional. Los resultados apuntan al déficit nutricional en las compras de alimentos de los hogares peruanos.

## Resumo

Evidências revelam uma relação direta entre a qualidade nutricional e os gastos com alimentação. No entanto, essas despesas são altamente suscetíveis a mudanças. Por outro lado, há uma limitação de evidências sobre a qualidade nutricional dos alimentos domésticos disponíveis. O objetivo deste estudo foi avaliar a relação entre a qualidade nutricional disponível e o gasto total com alimentos em domicílios peruanos, e se havia diferenças por área (urbana e rural) e entre os anos da pandemia de COVID-19. Para isso, foi utilizada a *Pesquisa Nacional de Domicílios do Peru* (ENAHO) de 2019 e 2020. Foram avaliados o gasto total com alimentos em dólares americanos por di e a qualidade nutricional disponível domiciliar com base na diversidade dietética e no cumprimento das necessidades calóricas domiciliares, considerando o percentual de gasto com alimentos e potenciais fatores de confusão. Utilizou-se o teste t de Student, a análise de variância (ANOVA), a regressão linear e o teste de Wald para avaliar o efeito de interação. Os domicílios com a qualidade nutricional disponível total/parcial adequada por área gastaram, em média, USD 2,00 a mais nas áreas urbanas do que nas rurais e tiveram um percentual de gasto com alimentos 7,1% maior em 2020 do que em 2019. Embora tenha sido encontrada uma associação entre a qualidade nutricional disponível e o gasto total com alimentos por ano e área de estudo, a modificação do efeito só estava presente por área de estudo. No modelo multivariável, os domicílios com a qualidade nutricional disponível total/parcial adequada apresentaram consistentemente um gasto total com alimentos menor por ano, assim como em áreas urbanas. Foi encontrada uma relação inversa entre a qualidade nutricional disponível e o gasto total com alimentos, em contraste com a relação direta dos estudos que avaliaram o custo da dieta e a qualidade nutricional. Esses resultados refletem o déficit nutricional nas compras de alimentos das famílias peruanas.

## Introduction

The causal determinants of adequate nutritional quality need to be explored worldwide for effective interventions ^1^. Recent evidence has concluded that adequate nutritional quality intake is strongly associated with high costs ^2^ ^,^ ^3^ ^,^ ^4^ ^,^ ^5^. Nevertheless, how the costs are estimated may directly impact these analyses ^6^ ^,^ ^7^ ^,^ ^8^. Usually, food prices have been used to calculate the dietary cost ^9^ ^,^ ^10^, but this could be affected by complex and dynamic mechanisms (i.e., trends, volatility, and spikes) ^11^. In contrast, total food expenditure, which determines selection patterns and purchasing strategies and can be independent of price elasticity ^12^ ^,^ ^13^, may be a good option. In fact, the causal directionality changes, making food expenditure a consequence of nutritional quality ^14^.

In nutritional sciences, food quality presents two different related concepts: the safety of food products ^15^ and the dietary content for a healthy status ^16^. The latter concept is related to food security, which measures nutritional quality by addressing the diversity and quantity of nutrients and how both can be affected by the availability, affordability, and utilization of food ^16^, characterizing the food purchasing considerations of the population ^17^. Indeed, food availability determines the quantity and quality of household food purchases ^18^. However, research is lacking to determine the factors that influence the available nutritional quality ^19^ ^,^ ^20^.

Despite studies on the association between nutritional quality intake and food expenditure existing, the analysis between available nutritional quality and total food expenditure has not yet been developed; therefore, we only know the relationship in behavior but not in the food choice ^21^ ^,^ ^22^. In Latin America, only Argentina has reported that households need to spend 32% more money on food to ensure a healthy diet, but this was based on the cost of the diet ^2^. In Peru, despite the dynamic economic growth in previous years, no evidence exists regarding the relationship between monetary factors and nutritional quality ^23^. However, 19% of the Peruvian population have shown to not have access to a healthy diet ^24^. Moreover, the number of malnourished Peruvians has increased due to the COVID-19 pandemic, similar to what has happened worldwide, reflecting a poor preventive and monitoring approach to food security ^25^. Therefore, this study aimed to assess the relationship between available nutritional quality and total food expenditure in Peruvian households and determine possible differences between urban and rural areas from 2019 to 2020. These two years were selected to evaluate the potential effect of the COVID-19 pandemic on the relationship of interest in a resource-constrained setting. We hypothesized that higher total food expenditure would be associated with higher adequate available nutritional quality, and more present in urban areas, higher in 2020 compared to 2019 due to the pandemic.

## Material and methods

### Study design

This is a secondary analysis using data from the *Peru’s National Household Survey* (ENAHO). The ENAHO is a multi-stage, stratified, nationally representative population-based survey conducted annually by the Peruvian National Institute of Statistics and Informatics (INEI). For this manuscript, data from 2019 and 2020 available on the INEI webpage (https://www.gob.pe/inei/) were used.

### Sample size

Currently, the ENAHO involves private households and their occupants residing in urban and rural areas of Peru. Subjects who are members of the Armed Forces living in barracks or similar, as well as those residing in collective dwellings (hotels, hospitals, asylums, convents, prisons, etc.) are usually excluded.

The ENAHO sampling is at a regional level with a three-stage approach. The primary sampling units are the population centers (rural with < 2,000 inhabitants and urban with ≥ 2,000 inhabitants). The secondary sampling units are clusters of 120 households on average. Finally, the third sampling unit consists of households since, in Peru, they are very dispersed in rural areas. In this manuscript, the unit of analysis is the household, considering the data of usual residents (i.e., including members of the family, domestic workers living inside the household, members of a family pension with < 10 pensioners, and people who are not members of the household but were present in the household in the 30 days before the survey). The survey is applied to the head of the household, spouse, persons aged ≥ 14 years old who receive monetary income, and individuals aged ≥ 12 years old.

For this analysis, we used the same selection criteria as those of the ENAHO but excluded data from households without information on food. Power calculations were estimated using Stata, version 17 (https://www.stata.com) considering the study by Marty et al. ^26^, which considers food expenditure data in their analysis of the real cost of the diet. The Satterthwaite’s t-test, with 0.05 significance level, 0.3 sample proportion, and 1,032 households, was used to obtain a power of greater than 80% to detect a difference of at least USD 0.54 expenditure per day (i.e., USD 4.44 vs. USD 4.98) between households with adequate and inadequate nutritional quality. With a design effect of 2, this sample size increased to 2,064, which was added to the ENAHO, with 2019 and 2020 data of 34,175 and 34,007 households, respectively. Therefore, this study provides accurate estimates, controlling for potential confounders and allowing comparison of results between subgroups (i.e., study year and study area) due to sample sizes.

### Definition of variables

The total food expenditure was the outcome of interest, defined as the amount of money spent per food item used in the household in the 15 days before the survey. Thus, the amount of money was added up considering all the food items reported and estimated in Peruvian currency. Then, this amount was converted to USD per day based on information from the Peruvian Central Reserve Bank (https://www.bcrp.gob.pe/), using average data of July for both 2019 and 2020.

The available nutritional quality was the exposure endpoint, defined based on the dietary diversity or calorie adequacy in the household. Dietary diversity was defined according to the variety of food available in the household at the time of ENAHO evaluation and based on 12 food groups (cereals; roots and tubers; vegetables; fruits; meat; eggs; fish and seafood; pulses, nuts, and seeds; milk and dairy products; oils and fats; sweets; and spices, condiments, and beverages). If any product of a food group was present at the time of the assessment, 1 point was summed to food diversity. Thus, a score from 0 to 12 was obtained. After that, the score was divided into three categories as low (≤ 3 points), medium (4 to 5), and high (≥ 6 points) ^27^.

Moreover, calorie adequacy estimates the energy balance between household needs as the total energy requirements of each household member divided by the total calorie availability. The total energy requirements were estimated for each household member according to age and sex, based on INEI recommendations for the Peruvian population ^28^; whereas the total calorie availability was determined by the calories for each food item added to the database according to each food reported from ENAHO. Data were collected from the Peruvian Table of Food Composition ^29^ and, if not available, from the Central American Table of Food Composition ^30^ or from the United States Department of Agriculture (USDA) ^31^.

For analysis, available nutritional quality was further classified as total or partial if the household dietary diversity was high/medium or calorie adequacy was from 90% to 110%. Otherwise (i.e., calorie adequacy was below 90% or above 110%), available nutritional quality was defined as inadequate.

Other variables were considered as potential confounders, including the average age of the family, the proportion of women in the household, and the number of children living in the household. This kind of analysis was possible since we had the characteristics of household members including sex (male or female) and age (in years). Other household characteristics included number of family members, geographical region (coast, highland, or jungle), and poverty level (extreme poor, non-extreme poor, or non-poor). The poverty level variable was extracted directly from the ENAHO database, which was defined based on the poverty line (i.e., minimum monetary value to determine whether the household is in a poverty situation). Moreover, we built a wealth index variable based on the criteria of the *Demographic and Health Survey* (DHS) of the United States Agency for International Development (USAID), calculated by the household’s ownership of certain assets and services ^32^. Moreover, the family income categorized by the minimum wage (PEN 930) was elaborated, and the percentage of food expenditure was developed by total food expenditure per 100 divided by total household expenditure. Finally, the study area (urban or rural) was considered as an effect modifier, as well as the years (2019 and 2020) due to the potential effect of the COVID-19 pandemic on the relationship of interest.

### Statistical analyses

The ENAHO database was used to obtain food availability and sociodemographic information. Data from 2019 and 2020 were used with differences expected between years. In 2019, a complete face-to-face measurement was conducted, whereas in 2020, almost half of the sample adopted a telephone interview modality, using a reduced questionnaire due to the COVID-19 pandemic. The database was downloaded, cleaned, and merged in Stata, version 17. Then, food data were coded, categorized, and had their calorie estimated. Foods not intended for human consumption, prepared (all culinary preparations), or unidentified, were associated to a particular code to exclude them from further analyses (Supplementary Material: https://cadernos.ensp.fiocruz.br/static//arquivo/suppl-e00021923_6884.pdf).

Multistage sampling was considered in the statistical analysis, using the *svy* command and the *subpop* option for subgroup analyses when required. A p-value < 0.05 was considered significant. Description of the study population was determined by the study year and using the Student’s t-test or analysis of variance (ANOVA) for numerical variables. Crude and adjusted linear regression models were created to estimate the association between the total food expenditure and available nutritional quality of the households. The interaction effect by year was evaluated in the crude and adjusted model using the Wald test. Therefore, the years 2019 and 2020 and rural and urban areas were evaluated separately to assess if differences occurred.

### Ethical approval

This study received approval from the Research Ethics Committee of the Southern Scientific University (Lima, Peru) (code 584-2021-POS50). Data is freely available on the INEI webpage without personal identifiers to guarantee participant confidentiality and anonymity.

## Results

### Characteristics of the study population

According to the study year, differences in the economic indicators were found, with an increase in poverty from 16% in 2019 to 23.2% in 2020 (p < 0.001). However, in the same lapse, an increase of 2.1% in the proportion of households with adequate total/partial available nutritional quality was also observed (p < 0.001). Moreover, no considerable change in total food expenditure was observed, being USD 5.30 (SD: 3.7) in 2019 and USD 5.20 (SD: 3.5) in 2020, but an increase in the percentage of food expenditure from 36.5% to 43.1% (p < 0.001) was found. On the other hand, the results show differences between rural and urban areas. Thus, poverty (i.e., extreme poor and non-extreme poor) was greater in rural areas (34.9%) compared to urban areas (15.4%, p < 0.001), but rural areas presented more households with adequate total/partial available nutritional quality (46.6%) compared to urban areas (29.5%, p < 0.001). Moreover, the total food expenditure in rural areas was lower compared to the urban area (USD 2.70 vs. USD 5.90, p < 0.001), although percentage of food expenditure was higher (17.6% vs. 15.8%, p < 0.001). Details are shown in Table 1.

**Table 1:** Characteristics of Peruvian households by study year (2019-2020) and study area (rural and urban).

	2019	2020	p-value	Rural	Urban	p-value
Characteristic of family members	n = 124,018	n = 121,800		n = 90,351	n = 155,467	
Number of family members [mean (SD)]	4 (2)	4 (2)	0.0092	3 (2)	4 (2)	< 0.0001
Age [mean (SD)]	34 (23)	33 (22)	0.0013	33 (23)	34 (22)	< 0.0001
Sex [n (%)]						
Female	63,500 (51.6)	62,078 (51.1)	0.0125	45,081 (50.4)	80,497 (51.6)	< 0.0001
Male	60,518 (48.4)	59,722 (48.9)		45,270 (49.6)	74,970 (48.4)	
Characteristic of households [n (%)]	n = 34,175	n = 34,007		n = 25,272	n = 42,910	
Geographical region						
Coast	14,166 (54.1)	14,333 (54.8)	0.4910	3,533 (10.5)	24,966 (66.8)	< 0.0001
Highland	13,177 (34.1)	12,833 (33.4)		15,388 (70.3)	10,622 (23.5)	
Jungle	6,832 (11.8)	6,841 (11.7)		3,533 (19.2)	7,322 (9.7)	
Wealth index						
Lowest	6,891 (13.7)	6,847 (14.7)	< 0.0001	13,839 (58.2)	2,135 (5.3)	< 0.0001
Second	6,830 (16.7)	6,759 (17.1)		2,104 (8.7)	965 (2.2)	
Middle	6,787 (20.3)	6,835 (21.8)		6,950 (25.7)	9,413 (21.8)	
Fourth	7,072 (25.1)	6,830 (23.6)		1,969 (6.2)	14,331 (34.1)	
Highest	6,595 (24.1)	6,736 (22.8)		410 (1.1)	16,066 (36.6)	
Poverty						
Extreme poor	888 (2.1)	1,388 (3.5)	< 0.0001	1,843 (8.4)	433 (1.3)	< 0.0001
Non-extreme poor	4,957 (13.9)	6,168 (19.7)		6,218 (26.5)	4,907 (14.1)	
Non-poor	28,330 (83.9)	26,451 (76.8)		17,211 (65.1)	37,570 (84.6)	
Family income (minimum wage)						
< 1	11,809 (28.6)	15,572 (42.0)	< 0.0001	16,261 (67.4)	11,120 (26.4)	< 0.0001
1 < 2	8,764 (25.2)	8,151 (25.1)		5,687 (21.1)	11,228 (26.3)	
2 < 3	5,254 (17.0)	4,329 (14.0)		1,907 (6.8)	7,676 (17.9)	
≥ 3	8,348 (29.2)	5,955 (18.9)		1,417 (4.7)	12,886 (29.4)	
Available nutritional quality						
Adequate total/partial	12,084 (32.2)	13,070 (34.3)	0.0001	11,839 (46.6)	13,315 (29.5)	< 0.0001
Inadequate	22,091 (67.8)	20,937 (65.7)		13,433 (53.4)	29,595 (70.5)	
Available dietary diversity						
Low	23,307 (71.4)	22,407 (69.7)	0.0009	14,165 (56.5)	31,549 (74.5)	< 0.0001
Medium	8,990 (24.2)	9,477 (25.3)		8,953 (35.6)	9,514 (21.7)	
High	1,878 (4.4)	2,123 (5.0)		2,154 (7.8)	1,847 (3.9)	
Energy requirement (± 10% adequacy)						
Adequate availability	1,589 (4.4)	1,984 (5.3)	0.0001	1,183 (5.1)	2,390 (4.8)	0.1453
Inadequate availability	32,586 (95.6)	32,023 (94.7)		24,089 (94.9)	40,520 (95.2)	
Characteristics of household spending [mean (SD)]						
Total food expenditure (USD per day) *	5.3 (3.7)	5.2 (3.5)	0.0297	2.7 (2.1)	5.9 (3.6)	< 0.0001
Percentage of food expenditure	36.5 (16.0)	43.1 (16.2)	< 0.0001	44.8 (17.6)	38.5 (15.8)	< 0.0001

SD: standard deviation.

* USD conversion is 3.52 for 2020 and 3.29 for 2019, according to the Peruvian Central Reserve Bank of Peru.

### Factors associated with total food expenditure

In the bivariable model, geographical region, wealth index, poverty, familial income, nutritional quality, dietary diversity, and energy requirement were associated with daily total food expenditure and percentage of food expenditure. The difference in daily total food expenditure was notable according to the study area, with the highest expenditure in urban areas. Compared to rural areas, households with adequate total/partial available nutritional quality in urban areas spent, on average, USD 2.00 more. Compared to rural households, urban households with high dietary diversity spent more (USD 0.90) as did those with adequate calorie availability (USD 3.60 more, p < 0.001). On the other hand, the difference in percentage of food expenditure was higher in 2020 compared to 2019 for: the percentage of food expenditure among households with adequate total/partial available nutritional quality were 44.1% vs. 37% (p < 0.001), 42.9% vs. 36.4% (p < 0.001) in households with high dietary diversity, and 47% vs. 40.5%, p < 0.001) in households with adequate calorie availability. Details are shown in Table 2.

**Table 2:** Factors associated with household total food expenditure and percentage of food expenditure, 2019-2020.

	2019	2020	p-value	Rural	Urban	p-value
	Mean (SD)	Mean (SD)		Mean (SD)	Mean (SD)	
Total food expenditure (USD per day) *						
Geographical region						
Coast	6.5 (3.3)	6.4 (3.1)	0.1569	4.8 (3.6)	6.4 (3.4)	< 0.0001
Highland	3.6 (3.1)	3.6 (2.9)	0.8583	2.4 (1.6)	4.7 (3.2)	< 0.0001
Jungle	4.4 (4.3)	4.6 (4.6)	0.1569	2.9 (2.4)	5.4 (4.9)	< 0.0001
Wealth index						
Lowest	2.5 (2.4)	2.7 (2.5)	< 0.0001	2.1 (1.6)	4.2 (2.9)	< 0.0001
Second	3.6 (2.9)	3.6 (2.8)	0.0467	2.3 (1.6)	4.9 (3.0)	< 0.0001
Middle	4.9 (2.9)	4.7 (2.8)	0.7612	2.5 (1.7)	5.9 (3.4)	< 0.0001
Fourth	6.0 (3.0)	6.0 (3.1)	0.7275	3.0 (2.2)	6.7 (3.5)	< 0.0001
Highest	7.9 (3.4)	7.6 (3.5)	0.0024	4.1 (3.0)	8.1 (4.0)	< 0.0001
Poverty						
Extreme poor	1.8 (1.5)	2.0 (1.7)	0.0001	1.4 (0.9)	2.8 (1.7)	< 0.0001
Non-extreme poor	3.5 (2.4)	3.9 (2.4)	< 0.0001	2.1 (1.3)	4.5 (2.3)	< 0.0001
Non-poor	5.8 (3.6)	5.6 (3.7)	0.4327	3.1 (2.4)	6.2 (3.8)	< 0.0001
Family income (minimum wage)						
< 1	2.4 (1.7)	3.2 (2.4)	< 0.0001	2.1 (1.4)	3.5 (2.3)	< 0.0001
1 < 2	4.7 (2.6)	5.2 (2.7)	< 0.0001	3.5 (2.1)	5.2 (2.7)	< 0.0001
2 < 3	6.3 (3.0)	6.6 (3.2)	< 0.0001	4.6 (2.8)	6.6 (3.1)	< 0.0001
≥ 3	8.1 (4.0)	8.5 (4.1)	0.0002	5.7 (3.7)	8.4 (4.0)	< 0.0001
Nutritional quality						
Inadequate	6.0 (3.8)	5.9 (3.6)	0.0362	3.0 (2.4)	6.6 (3.7)	< 0.0001
Adequate total/partial	3.8 (2.9)	3.8 (2.9)	0.1561	2.4 (1.7)	4.4 (3.0)	< 0.0001
Dietary diversity						
Low	6.0 (3.9)	5.9 (3.6)	0.0377	3.0 (2.4)	6.6 (3.7)	< 0.0001
Medium	3.5 (2.4)	3.6 (2.5)	0.0717	2.4 (1.6)	4.1 (2.5)	< 0.0001
High	2.6 (2.0)	2.7 (1.9)	0.1259	2.1 (1.3)	3.0 (2.0)	< 0.0001
Energy requirement (± 10% adequacy)						
Inadequate availability	5.2 (3.7)	5.1 (3.5)	0.0288	2.7 (2.1)	5.9 (3.6)	< 0.0001
Adequate availability	6.3 (4.2)	6.1 (4.0)	0.1478	3.4 (2.2)	7.0 (4.2)	< 0.0001
Percentage of food expenditure						
Geographical region						
Coast	34.2 (12.7)	41.7 (13.5)	< 0.0001	46.3 (18.7)	37.7 (14.3)	0.0001
Highland	39.6 (18.5)	45.4 (18.2)	< 0.0001	45.3 (16.6)	40.2 (17.4)	< 0.0001
Jungle	38.1 (21.2)	43.2 (21.9)	< 0.0001	42.1 (19.8)	39.9 (21.7)	< 0.0001
Wealth index						
Lowest	44.9 (21.9)	49.0 (20.7)	< 0.0001	45.9 (19.4)	45.4 (16.6)	0.0092
Second	42.6 (18.0)	48.5 (17.8)	< 0.0001	47.6 (16.8)	41.6 (15.7)	0.0240
Middle	38.4 (15.3)	45.2 (15.0)	< 0.0001	45.2 (16.1)	38.7 (14.9)	0.0019
Fourth	34.6 (12.6)	42.8 (13.6)	< 0.0001	44.3 (16.8)	35.9 (14.0)	0.0007
Highest	27.9 (10.5)	35.4 (12.8)	< 0.0001	39.7 (18.0)	29.9 (12.9)	0.3214
Poverty						
Extreme poor	44.8 (18.8)	48.2 (17.0)	0.0002	45.9 (16.2)	48.8 (12.5)	0.0049
Non-extreme poor	45.0 (15.4)	49.0 (14.0)	< 0.0001	46.9 (16.3)	47.6 (12.4)	0.1366
Non-poor	34.9 (15.5)	41.4 (16.3)	< 0.0001	44.8 (18.2)	36.8 (15.9)	< 0.0001
Family income (minimum wage)						
< 1	44.7 (17.7)	48.7 (16.2)	< 0.0001	47.8 (17.3)	46.6 (16.7)	0.0001
1 < 2	38.4 (14.9)	43.4 (15.2)	< 0.0001	40.7 (16.6)	40.9 (14.9)	0.4253
2 < 3	34.3 (13.1)	38.7 (14.1)	< 0.0001	36.2 (15.7)	36.8 (13.6)	0.2582
≥ 3	28.2 (11.7)	33.0 (13.3)	< 0.0001	32.6 (15.2)	30.0 (12.4)	< 0.0001
Nutritional quality						
Inadequate	36.3 (15.1)	42.6 (15.4)	< 0.0001	43.7 (17.8)	38.5 (15.2)	< 0.0001
Adequate total/partial	37.0 (17.9)	44.1 (17.7)	< 0.0001	46.0 (17.3)	38.3 (17.2)	< 0.0001
Dietary diversity						
Low	36.5 (15.1)	42.9 (15.5)	< 0.0001	44.1 (17.7)	38.7 (15.3)	< 0.0001
Medium	36.7 (17.8)	43.9 (17.7)	< 0.0001	45.7 (17.2)	38.0 (17.0)	< 0.0001
High	36.4 (20.7)	42.9 (19.5)	< 0.0001	45.7 (18.2)	36.6 (18.9)	< 0.0001
Energy requirement (± 10% adequacy)						
Inadequate availability	36.3 (15.9)	42.9 (16.2)	< 0.0001	44.5 (17.7)	38.3 (15.8)	< 0.0001
Adequate availability	40.5 (16.4)	47.0 (16.4)	< 0.0001	50.9 (15.1)	42.0 (16.9)	< 0.0001

SD: standard deviation.

* USD conversion is 3.52 for 2020 and 3.29 for 2019, according to the Peruvian Central Reserve Bank of Peru.

### Association between available nutritional quality and total food expenditure

An association between available nutritional quality and total food expenditure was found by year and study area. Study area was an effect modifier of the association in adjusted models, but it was not the case for study year. In the multivariable model, households with adequate total/partial available nutritional quality and with medium or high dietary diversity presented lower total food expenditure and percentage of food expenditure, and this finding was consistent by year. However, households with adequate caloric availability demonstrated higher total food expenditure and percentage of food expenditure (Table 3). By study area, the total food expenditure for households with adequate total/partial available nutritional quality was significantly lower in urban compared to rural areas. Nevertheless, in 2020, households with adequate total/partial available nutritional quality in rural areas presented higher percentage of food expenditure compared to those with inadequate available nutritional quality, whereas urban area households with adequate total/partial available nutritional quality demonstrated lower percentage of food expenditure compared to those with inadequate available nutritional quality (Table 4).

**Table 3:** Food expenditure and available nutritional quality at household level, 2019-2020.

	2019				2020			
	Crude model		Adjusted model *		Crude model		Adjusted model *	
	β	95%CI	β	95%CI	β	95%CI	β	95%CI
Total food expenditure (USD per day) **								
Nutritional quality								
Inadequate	1.00	Reference	1.00	Reference	1.00	Reference	1.00	Reference
Adequate total/partial	-2.23	-2.33; -2.13	-0.82	-0.88; -0.76	-2.03	-2.14; -1.93	-0.78	-0.84; -0.72
Dietary diversity								
Low	1.00	Reference	1.00	Reference	1.00	Reference	1.00	Reference
Medium	-2.56	-2.66; -2.45	-1.28	-1.36; -1.20	-2.34	-2.46; -2.23	-1.14	-1.23; -1.06
High	-3.44	-3.59; -3.28	-1.73	-1.87; -1.59	-3.19	-3.32; -3.06	-1.59	-1.72; -1.47
Energy requirement (± 10% adequacy)								
Inadequate availability	1.00	Reference	1.00	Reference	1.00	Reference	1.00	Reference
Adequate availability	1.08	0.81; 1.35	1.31	1.13; 1.49	0.92	0.69; 1.16	1.09	0.91; 1.26
Percentage of food expenditure								
Nutritional quality								
Inadequate	1.00	Reference	1.00	Reference	1.00	Reference	1.00	Reference
Adequate total/partial	0.68	0.18; 1.18	-1.57	-1.90; -1.24	1.49	0.95; 2.02	-0.69	-1.03; -0.35
Dietary diversity								
Low	1.00	Reference	1.00	Reference	1.00	Reference	1.00	Reference
Medium	0.20	-0.36; 0.76	-3.32	-3.80; -2.83	1.06	0.47; 1.66	-2.05	-2.61; -1.48
High	-0.05	-1.32; 1.22	-4.98	-6.04; -3.91	0.06	-1.07; 1.20	-4.13	-5.17; -3.08
Energy requirement (± 10% adequacy)								
Inadequate availability	1.00	Reference	1.00	Reference	1.00	Reference	1.00	Reference
Adequate availability	4.15	3.08; 5.23	4.52	3.64; 5.41	4.12	3.09; 5.14	4.61	3.76; 5.46

95%CI: 95% confidence interval.

Note: independent regressions, each variable analyzed separately.

* Adjusted by familial income, wealth index score, geographical region, number of children in the household, proportion of women in the household, and average family age;

** USD conversion is 3.52 for 2020 and 3.29 for 2019, according to the Peruvian Central Reserve Bank.

**Table 4:** Food expenditure and available nutritional quality at household level by area in 2019-2020.

	Rural				Urban			
	Crude model		Adjusted model *		Crude model		Adjusted model *	
	β	95%CI	β	95%CI	β	95%CI	β	95%CI
Total food expenditure (USD per day) *								
2019								
Nutritional quality								
Inadequate	1.00	Reference	1.00	Reference	1.00	Reference	1.00	Reference
Adequate total/partial	-0.62	-0.71; -0.52	-0.31	-0.37; -0.24	-2.26	-2.39; -2.13	-1.16	-1.27; -1.05
2020								
Nutritional quality								
Inadequate	1.00	Reference	1.00	Reference	1.00	Reference	1.00	Reference
Adequate total/partial	-0.56	-0.66; -0.46	-0.23	-0.30; -0.15	-2.02	-2.15; -1.89	-1.02	-1.13; -0.91
Percentage of food expenditure								
2019								
Nutritional quality								
Inadequate	1.00	Reference	1.00	Reference	1.00	Reference	1.00	Reference
Adequate total/partial	1.60	0.89; 2.30	0.50	-0.15; 1.16	-1.02	-1.64; -0.41	-3.22	-3.78; -2.67
2020								
Nutritional quality								
Inadequate	1.00	Reference	1.00	Reference	1.00	Reference	1.00	Reference
Adequate total/partial	2.61	1.87; 3.35	1.61	0.90; 2.32	0.25	-0.42; 0.92	-2.06	-2.69; -1.43

95%CI: 95% confidence interval.

Note: independent regressions, each variable analyzed separately.

* Adjusted by familial income, wealth index score, geographical region, number of children in the household, proportion of women in the household, and average family age;

** USD conversion is 3.52 for 2020 and 3.29 for 2019, according to the Peruvian Central Reserve Bank.

## Discussion

Our results show that households with adequate partial/total available nutritional quality presented lower total food expenditure, and this relationship was observable by year (2019 and 2020) and by area (rural and urban). In addition, only the study area (i.e., rural vs. urban) was an effect modifier of the relationship between available nutritional quality and total food expenditure, implying a different purchasing pattern for food choice by study area ^6^ ^,^ ^33^. Households with higher dietary diversity showed lower money expenditure per day and percentage points compared with households with poor diversity, whereas households that met the caloric requirements presented higher money expenditure per day and percentage points compared with households that did not.

The percentage of food expenditure increase from 2019 to 2020 that we found is in line with Engel’s law that “*in poorer households: total food expenditure has a lower absolute value, but a higher proportion*” ^34^ (p. 4). Some studies with a similar methodology have reported similar results, in which the total food expenditure among households with higher economic income doubled that of households with a lower income ^35^ ^,^ ^36^. On the other hand, our study showed that the total food expenditure is three times higher in urban than in rural areas, which contrasts with the pattern of other research using a nationally representative household economic survey, showing a lower total food expenditure in more urbanized areas ^37^. However, the pattern reported in the latter study was lost in some rural areas due to the appropriate use of natural resources, reducing the total food expenditure ^37^. This is consistent with the characteristics of rural areas in Peru, where there is greater access to agricultural food, practices of food self-sufficiency, trading, and lower food prices on average ^38^.

While many studies have found a direct association between dietary cost and nutritional quality ^2^ ^,^ ^6^ ^,^ ^39^ ^,^ ^40^, our study focused on using the total food expenditure instead of dietary costs. The decision to use the total food expenditure was due to limitations of dietary cost, such as overestimation due to possible errors in the reporting of price ^26^ ^,^ ^41^. Thus, studies using the total food expenditure can capture the variability of purchasing choices in the population ^13^, obtaining an inverse relationship with indicators of diet quality ^22^. In this study, the inverse relationship obtained between total food expenditure and available nutritional quality shows that Peruvian households prioritizing lower-cost foods can obtain greater diversity, but the amount of total food expenditure invested in food is not sufficient to cover 90% to 110% of calorie adequacy. Since our study is based on household food availability, these results may be due to the high proportion of out-of-home consumption ^42^. In 2019, the average expenditure of food consumption outside the household was four times higher than inside the household. By contrast, in 2020, the average expenditure outside the household was three times lower than inside the household.

In our study, the survey year (i.e., 2019 vs. 2020) was not an effect modifier, which is consistent with an analysis of food expenditure in the first year of the pandemic, in which no visible effect of the total food expenditure available to the household was noted ^43^. This latter analysis was performed using national surveys with a similar methodology as that used in the present work. On the other hand, the study area was an effect modifier of the association of interest. Thus, the difference in total food expenditure obtained by households with adequate total/partial available nutritional quality compared with households with inadequate available nutritional quality is lower in rural than urban areas. Other studies with a similar methodology have shown that agricultural production occurs in rural areas and the food products are destined for urban areas and, as a result, households located in rural areas do not consume their own products, generating a higher total food expenditure ^44^ ^,^ ^45^. Instead, the change to a direct relationship between total food expenditure and available nutritional quality obtained by rural areas in 2020 is a reflection of the greater impact of the COVID-19 pandemic in this area. Our results show that households with adequate total/partial available nutritional quality spend USD 0.23 less per day than households with inadequate available nutritional quality ^46^.

Our findings, which show that households with adequate total/partial available nutritional quality present lower total food expenditure, may reflect poor household food shopping strategies for greater variety, implying a more monotonous and more expensive diet, maybe with a greater presence of ultra-processed foods. Moreover, the implementation of self-sufficiency techniques would be of interest to reduce the total food expenditure in rural areas ^47^. Furthermore, our descriptive results show that more than half of Peruvian families do not have enough food to satisfy the nutritional requirements of all the household members. Only 5% meet the availability of diverse foods and sufficient calories, regardless of the impact of the COVID-19 pandemic. Therefore, it is advisable to evaluate the quality of current nutritional programs to adjust them to the needs of the vulnerable population. In addition, further studies should be conducted to evaluate behavioral-economic strategies such as marketing evaluation, nutrition labeling, and octagons and their impact on food preferences ^48^. Furthermore, the use of the ENAHO database can be an annual indicator to monitor improvements in dietary quality in Peru and its relationship with total food expenditure ^49^.

The major strength of this study is the use of information from two consecutive years of a nationally representative database. Moreover, the instruments used for measurement present high reliability and validity. However, limitations in our study deserve discussion. First, the lack of standardization of food denomination in the ENAHO database could be a concern since they may result in the loss of relevant nutritional information for the analysis, such as misspelled commonly consumed foods that could not be identified. However, the percentage of foods not considered (non-recognition 0.77% and 0.67%, non-human consumption 0.78% and 0.6%, culinary preparations 2.42% and 2.3%, in 2019 and 2020 respectively) was similarly affected by area and year. Second, the lack of nutritional information for several foods consumed in the country can be a problem. The use of external nutritional information such as the USDA food database and the Central American Table of Food Composition was in 30% of foods and this may affect our results. Third, we found foods of non-monetary origin (such as donations, exchanges within the community, from their own harvest/farm) without complementary information to know the percentage of purely monetary origin. However, this represented only 3.6% and 5.5% of the total food assessed in 2019 and 2020, respectively, a minor proportion which likely has a negligible effect compared to the amount of similar food assessed of purely monetary origin. Fourth, the 2020 ENAHO data may be affected by the COVID-19 pandemic, as 49% of the total sample adopted the telephone interview modality with a summary questionnaire as a preventive measure. However, the results were similar in quality to those obtained in 2019, which we believe may not affect the study’s conclusions. Finally, the analyses focused on assessing only foods available in the household from the information in the basic food basket; therefore, no inferences can be made on food consumption or food available in general.

## Conclusion

Peruvian households with adequate total/partial available nutritional quality have a lower total food expenditure, and those with high or medium diversity have lower expenditure than those with low diversity. However, an increase in total food expenditure is needed to obtain caloric adequacy. These results did not seem to differ due to the COVID-19 pandemic; however, rural area households present lower differences in total food expenditure for households with adequate total/partial available nutritional quality and inadequate available nutritional quality, whereas urban area households present higher differences.
